# Radiofrequency Ablation for the Treatment of Hepatocellular Carcinoma Just Below the Hepatic Capsule

**DOI:** 10.3390/cancers18203346

**Published:** 2026-10-16

**Authors:** Takashi Tanaka, Kazuhide Takata, Yuya Sasahara, Yasuharu Shimoji, Hiroaki Tokushige, Takahiro Nagata, Takashi Miyayama, Youtaro Uchida, Kumiko Shibata, Hiromi Fukuda, Ryo Yamauchi, Keiji Yokoyama, Fumihito Hirai

**Affiliations:** Department of Gastroenterology and Medicine, Faculty of Medicine, Fukuoka University, Nanakuma 7-45-1, Fukuoka 814-0180, Japan; edihuzak_t@yahoo.co.jp (K.T.); y.sasahara.dy@adm.fukuoka-u.ac.jp (Y.S.); y.shimoji.le@adm.fukuoka-u.ac.jp (Y.S.); h.tokushige.rs@adm.fukuoka-u.ac.jp (H.T.); nagata0529@adm.fukuoka-u.ac.jp (T.N.); tmiyayama@adm.fukuoka-u.ac.jp (T.M.); yoota86@adm.fukuoka-u.ac.jp (Y.U.); kumikoshibata@fukuoka-u.ac.jp (K.S.); hfukuda@fukuoka-u.ac.jp (H.F.); r_christinue2017@yahoo.co.jp (R.Y.); yokotin@fukuoka-u.ac.jp (K.Y.); fuhirai@cis.fukuoka-u.ac.jp (F.H.)

**Keywords:** hepatocellular carcinoma, locoregional treatment, radiofrequency ablation, subcapsular HCC, local tumor progression, tumor seeding

## Abstract

**Background/Objectives**: During radiofrequency ablation (RFA) for the treatment of hepatocellular carcinoma (HCC), electrodes are generally inserted into the center of the tumor through the hepatic parenchyma under image guidance, including ultrasound or computed tomography (CT) guidance. However, tumors located just below the hepatic capsule require special puncture techniques. This retrospective study aimed to analyze the efficacy and outcomes of RFA for HCC located just below the subcapsular region of the liver. **Methods**: A total of 81 patients with 81 subcapsular HCC tumors underwent RFA. Baseline patient characteristics, treatment results, and adverse events were investigated. Factors associated with local recurrence were also examined. **Results**: Among the 81 patients, 59 (73%) were male, the median age was 74 years (range, 46–93 years), and 68 (84%) were classified as Child-Pugh class A. The median tumor size was 1.5 cm (range, 0.5–3.7 cm), and 56 of 81 tumors (69%) were <2.0 cm in diameter. 53 tumors were located in the right lobe of the liver (65%). Monopolar RFA was performed in 55 patients (68%), and no-touch multi-bipolar RFA was performed in 26 patients (32%). Treatment success was achieved in 75 tumors (93%), with inadequate ablative margins observed in 6 tumors (7%). Of these six tumors, five (83%) were treated with monopolar RFA; however, differences between the RFA devices were not statistically significant (*p* = 0.368). Local tumor progression (LTP) was observed in 19 patients (24%), with cumulative LTP rates of 13%, 29%, 33%, and 33% at 1, 3, 5, and 10 years, respectively. Multivariate analysis using a COX proportional hazards model identified insufficient ablative margins as a significant factor associated with LTP (hazard ratio, 4.64; 95% confidence interval, 1.14–14.9; *p* = 0.031). Adverse events occurred in 17 patients (21%), all of whom recovered with conservative treatment. Tumor seeding occurred in only one patient (1%). **Conclusions**: In the present study, achieving an adequate ablative margin using either monopolar or multi-bipolar RFA systems was associated with a reduced risk of local tumor recurrence in patients with subcapsular HCC.

## Simple Summary

Radiofrequency ablation (RFA) is a minimally invasive treatment for patients with early-stage hepatocellular carcinoma (HCC). However, HCC located just beneath the liver capsule can be technically challenging to treat because of its close proximity to surrounding organs and the risk of complications such as bleeding, thermal injury, and tumor seeding. Although previous studies have reported favorable outcomes of RFA for subcapsular HCC, evidence regarding its long-term effectiveness and safety remains limited. In this study, we evaluated the clinical outcomes and complications of RFA for subcapsular HCC based on our institutional experience. We also examined technical factors that may affect treatment outcomes, including the adequacy of the ablative margin and the RFA procedure. Our findings may help clarify the safety and effectiveness of RFA for subcapsular HCC and provide practical guidance for performing RFA in this challenging anatomical location.

## 1. Introduction

Hepatocellular carcinoma (HCC) is the most common primary malignancy of the liver and represents a major global health burden, particularly in patients with underlying chronic liver disease or cirrhosis [1,2]. Among various treatment modalities, radiofrequency ablation (RFA) has emerged as a minimally invasive, curative-intent approach for patients with early-stage HCC who are unsuitable for surgical resection or liver transplantation [3]. A Japanese multicenter prospective study (SURF study) comparing RFA treatment with surgical treatment for early-stage HCC reported that the survival rate was equivalent to that of resection, establishing RFA as a curative treatment for early-stage HCC [4]. However, some early-stage HCCs are unsuitable for or difficult to treat with RFA. Percutaneous RFA has not been able to treat liver nodules that are difficult to visualize using ultrasound, located behind large blood vessels, or in contact with organs surrounding the liver, such as the heart, intestinal tract, gallbladder, and lungs, where RFA treatment is expected to damage other organs. Although CT-guided RFA or microwave ablation (MWA) has been reported to achieve a high technical success rate and adequate ablative margins, particularly for HCCs in anatomically challenging locations [5,6], its application may be limited by technical complexity and increased procedural costs. They are often treated with hepatic resection, transcatheter arterial chemoembolization (TACE), percutaneous ethanol injection therapy (PEIT), or pharmacotherapy instead of ablation therapy [3]. Tumors located in the hepatic capsule, particularly those abutting the liver surface, present unique technical challenges and risks during RFA [7]. The proximity to adjacent structures such as the diaphragm, gastrointestinal tract, or abdominal wall increases the risk of complications such as bleeding, thermal injury to neighboring organs, and insufficient ablative margins [8]. Furthermore, subcapsular HCC may be more prone to post procedural complications, including peritoneal seeding or bleeding. Despite these challenges, recent advances in imaging guidance, electrode design, and procedural planning have expanded the applicability of RFA to challenging anatomical locations. However, the efficacy and safety of RFA for subcapsular HCC remain an area of active investigation. Although several studies have reported good outcomes for the treatment of subcapsular HCC with RFA [7,9,10,11,12], studies comparing various treatment modalities or establishing a sufficient observation period have not been conducted. The aim of this study was to evaluate the clinical outcomes, technical considerations, and complication profiles associated with RFA for subcapsular HCC, by drawing on the experience in our institution.

## 2. Materials and Methods

### 2.1. Study Design

**Figure 1:**
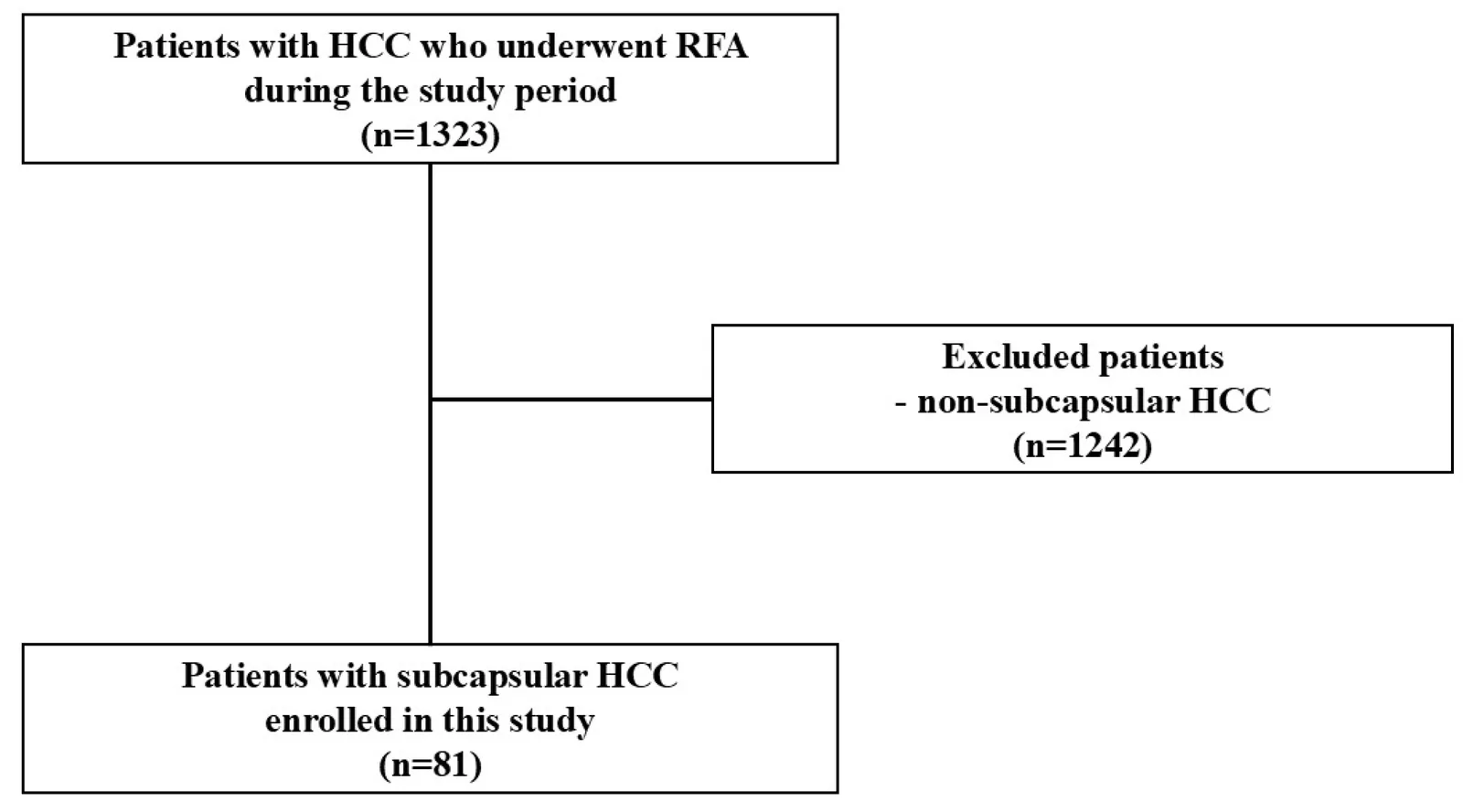
Patient’s selection in this study.

This retrospective study was conducted between January 2009 and October 2022 in the Department of Gastroenterology and Medicine at our institution. During the study period, 1323 patients with early-stage HCC underwent RFA, and 81 subcapsular HCC cases were selected based on the database (Figure 1). In this study, we defined subcapsular tumor as an HCC nodule abutting the superficial liver capsule that could not be directly punctured using percutaneous ultrasound guidance. This study was approved by the Institutional Review Board (approval no. H23-09-003) and was conducted in accordance with the principles of the Declaration of Helsinki. Written informed consent was obtained from all patients prior to RFA.

### 2.2. Endpoints

The primary endpoint was the rate of local tumor progression (LTP), and the secondary endpoints were adverse events and tumor seeding caused by RFA treatment of subcapsular HCC. LTP was defined as recurrence of HCC at or adjacent to the margin of the previously treated ablation zone and refers to reappearing viable tumor provided that at least one contrast-enhanced follow-up study did not reveal residual viable tumor at the ablative margin [13]. Adverse events were evaluated by Clavien–Dindo classification. Additionally, the prognostic factors associated with LTP in patients who underwent RFA for subcapsular HCC were investigated using multivariate analysis.

Selection criteria for RFA: This study included 81 patients with subcapsular HCC located just beneath the hepatic capsule. Contrast-enhanced dynamic computed tomography (CT) scan and/or magnetic resonance imaging (MRI) were performed within one month before RFA. Based on the American Association for the Study of Liver Diseases practice guidelines for HCC management, HCC was diagnosed when the lesion displayed hypervascularity in the arterial phase and washout in the portal venous or delayed phase on dynamic CT and/or MRI [1]. Subcapsular HCC was defined as a tumor that could not be directly punctured under percutaneous ultrasound (US) guidance owing to its contact with the liver surface on US images. For example, nodules that were in contact with the liver surface but were located on the posterior aspect and could be punctured by using conventional methods were excluded. The following variables were analyzed: age, sex, etiology, tumor diameter, number of tumors, number of punctures, use of monopolar or bipolar electrodes, and post-puncture margins. Exclusion criteria included age < 18 years, pregnancy, platelet count < 50,000/μL, hepatic reserve Child-Pugh class C, patients with bleeding tendency (e.g., hemophilia or coagulopathy), and ongoing treatment for other malignancies.

### 2.3. RFA Procedure and Follow-Up

**Figure 2:**
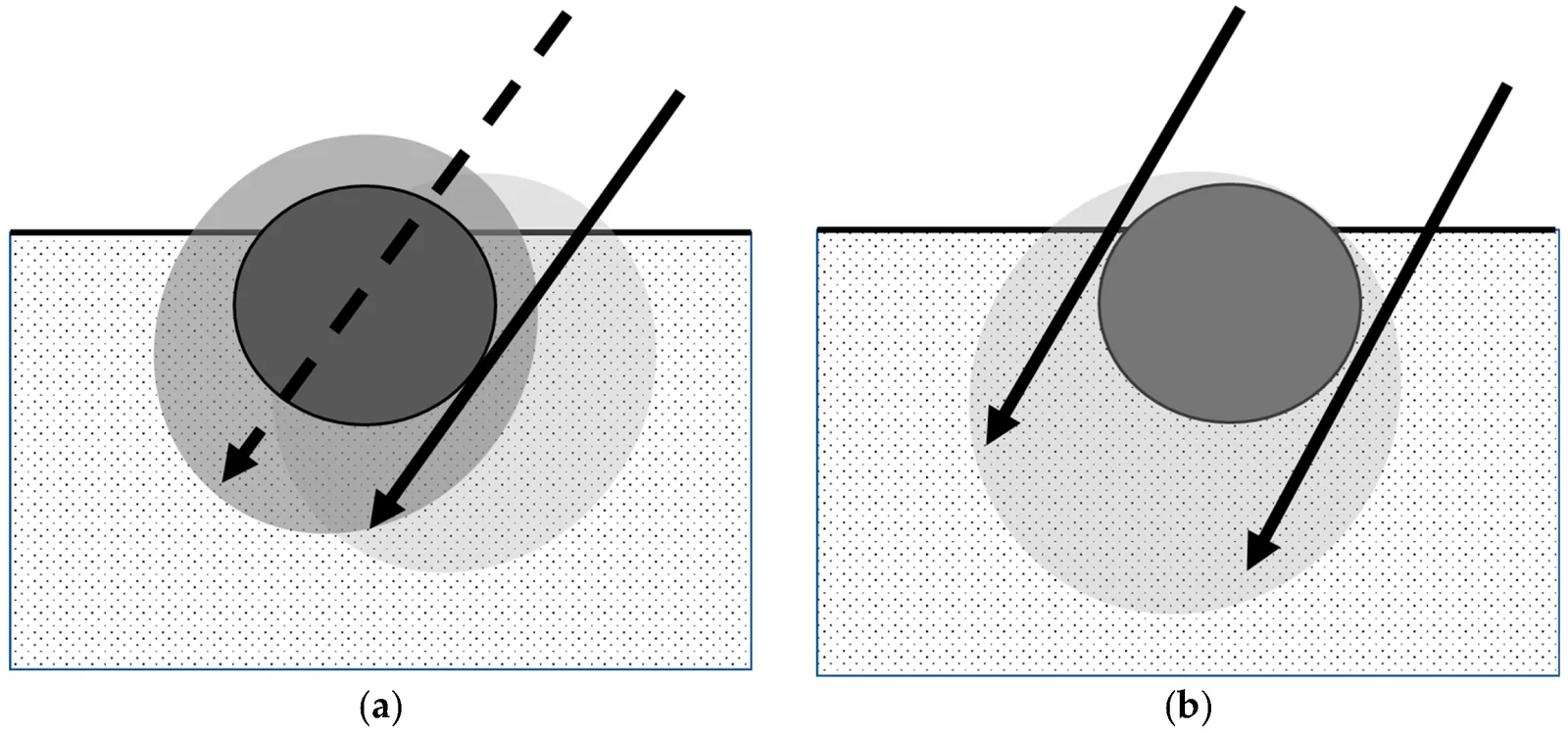
RFA technique for hepatocellular carcinoma located at the hepatic capsule. (**a**) RFA with a monopolar electrode. First, the electrode was inserted outside of the tumor via the liver parenchyma (solid line). An additional ablation session was performed if the bubble did not cover the tumor. In the second or subsequent sessions, the electrode was punctured directly into the tumor, which seemed to be ablated (dashed line). (**b**) No-touch multi-bipolar RFA was performed from 2014. Several bipolar electrodes were inserted surrounding the tumor and not punctured directly into the tumor. The oval-shaped grey area in the both figure (**a**,**b**) represents the ablative area.

The puncture technique used for subcapsular HCC in this study is shown in Figure 2. In all cases, the procedure was performed percutaneously under US guidance and local anesthesia. The electrode needle was inserted into the lateral side of the nodule instead of being directly inserted into the nodule, to avoid bleeding and seeding. Once the generated ablation bubbles sufficiently encompassed the entire nodule, the procedure was completed in a single session. However, if the ablation bubble did not adequately cover the nodule, which was considered insufficient treatment, additional cauterization was performed using two or more insertion routes (Figure 2a). A monopolar RFA system was used, which involved the insertion of a 17-gauge internally cooled electrode with a 20- or 30 mm-long exposed tip (Cool-tip; Covidien, Boulder, CO, USA), and a 17-gauge internally cooled electrode with 10-, 15-, 20-, 25-, and 30 mm variable exposed tips (VIVA RF System; Starmed, Goyang-si, Gyeonggi-do, Republic of Korea). The Cool-Tip System was used from 2009 to 2017, and the VIVA RFA system was used from 2017 to 2022. A multi-bipolar RFA system with bipolar internally cooled electrodes (Celon Lab Power, Olympus Corporation, Tokyo, Japan) was available from 2014 to 2022. Multi-bipolar RFA treatment was performed using the no-touch ablation method, in which electrode needles were inserted into both sides of the nodule to achieve ablation without direct tumor puncture (Figure 2b). Percutaneous RFA was performed by physicians with ≥5 years of experience in RFA of liver tumors at our institute, and the selection of RFA devices was based on the operator’s decision. Radiofrequency energy was delivered via a generator using an impedance-based control algorithm, according to the manufacturer’s instructions. In all cases, tract ablation was performed when the electrode needle was removed, and the treatment was completed after confirming the absence of bleeding. After RFA, treatment efficacy was evaluated using CT within 3 days. Treatment efficacy was considered sufficient when the ablative area had a margin of at least 5 mm beyond the tumor edge, as assessed by CT or MRI after RFA. Thereafter, the patients were followed up with CT or MRI every 3 months.

### 2.4. Additional Supporting Treatment Before RFA

Several supporting techniques were used to enhance the safety and effectiveness of RFA treatment for subcapsular HCC. TACE was performed within 1–2 weeks before RFA for tumors with a diameter > 2.0 cm. PEIT was performed just before starting RFA for tumors protruding outside the liver. A 21-gauge PEI needle (Hakko, Nagano, Japan) or 22-gauge PTCD needle (Top, Tokyo, Japan) was inserted at the tumor edge. Absolute ethanol was slowly injected into the tumor and ablation was performed after the PEI needle was withdrawn. Artificial ascites with a 50% glucose solution was induced when required to improve the US window and decrease thermal injury to the adjacent diaphragm, lungs, heart, gallbladder, stomach, and colon.

### 2.5. Statistical Analysis

The LTP rate was analyzed using the Kaplan–Meier method, and the differences between the curves were assessed using the log-rank test. Independent predictive factors associated with LTP were identified using multivariate Cox proportional hazards regression analysis. We defined the number of covariates included in the multivariable analysis by selecting clinically relevant variables and variables showing a potential association with LTP in the univariate analysis. Statistical analyses were conducted using the JMP software for Windows, version 14.2.0 (SAS Institute, Cary, NC, USA). Differences with *p* value < 0.05 were considered statistically significant.

## 3. Results

### 3.1. Patient Background

Among 1323 patients with HCC who underwent RFA during the study period, 81 patients with subcapsular HCC, involving a total of 81 tumors, were enrolled in this study. Patient characteristics are shown in Table 1. The median age was 74 years, and 59 patients (73%) were male. Sixty-eight patients (84%) were classified as Child-Pugh class A, and 21 (26%) had treatment-naïve HCC. The median AFP level was 6.9 ng/mL (range: 1.1–280 ng/mL), and the median DCP level was 27 mAU/mL (range: 10–3755 mAU/mL). The median tumor size was 1.5 cm in diameter (range: 0.5–3.7 cm). The tumor was located in the right hepatic lobe in 53 patients (65%) and the left lobe in 28 patients (35%). None of the tumors were located in the caudate lobe.

### 3.2. RFA Procedures and Technical Efficacy

The treatment characteristics of RFA are presented in Table 2. Fifty-five patients (68%) underwent monopolar RFA, and 26 patients (32%) received multi-bipolar RFA. All patients underwent a single RFA procedure. Additional treatments before RFA included TACE in 10 patients (12%) and PEIT in seven patients (9%). Two patients had undergone both TACE and PEIT prior to RFA. Artificial ascites was used in 26 cases (32%) to facilitate the procedure. The number of electrode insertions was as follows: one in 34 cases (42%), two in 33 cases (41%), three in 12 cases (15%), and five in two cases (2%). None of the patients required four insertions. Adequate ablative margins (>5 mm) were achieved in 75 patients (93%). The median follow-up period was 27 months (range, 3–157 months).

### 3.3. Local Tumor Progression Rate

**Figure 3:**
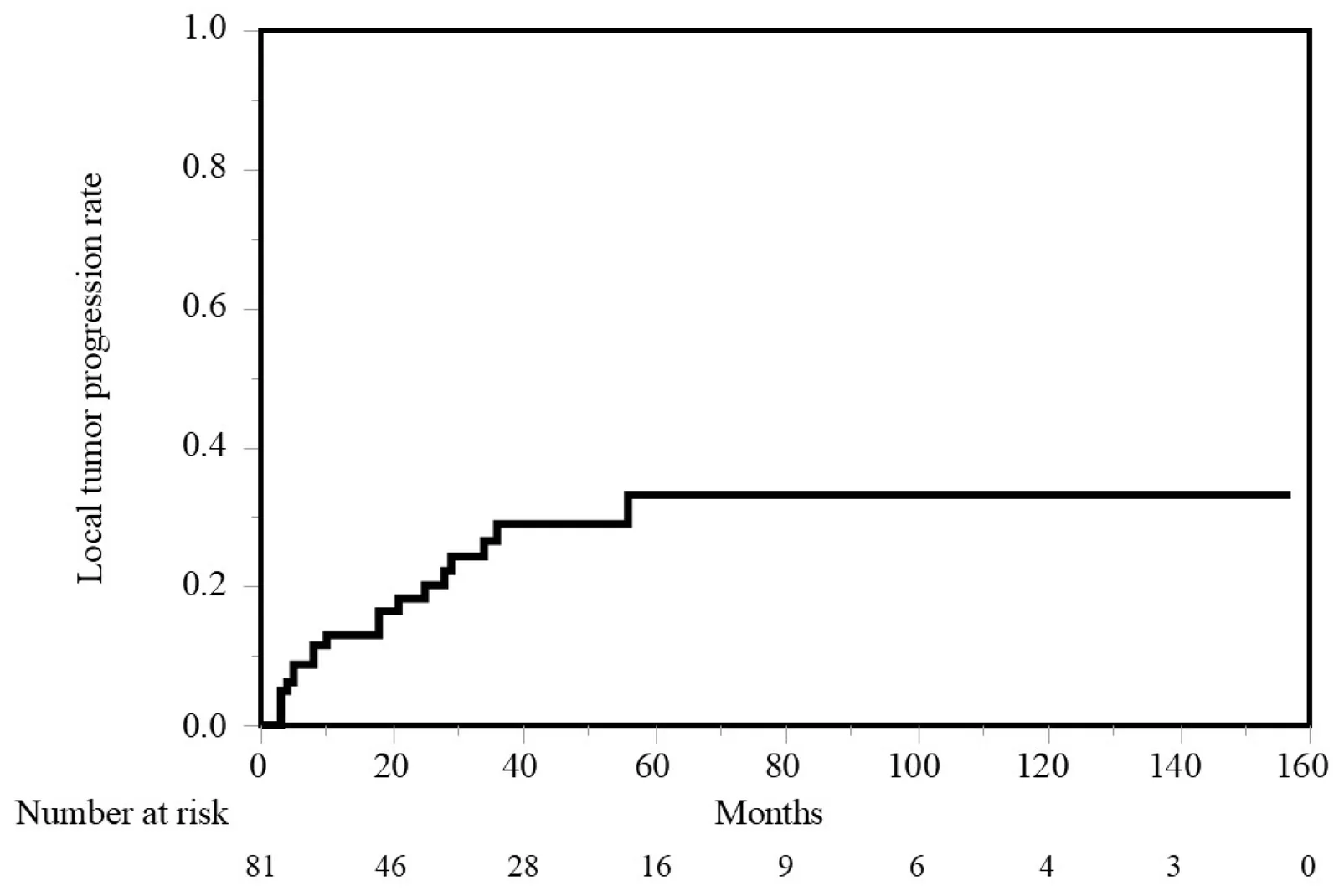
Kaplan–Meier curves for local tumor progression in this study population. Local tumor progression was observed in 19 patients; the local tumor recurrence rate was 13% at 1 year, 29% at 3 years, 33% at 5 years, and 33% at 10 years after initial RFA treatment.

**Figure 4:**
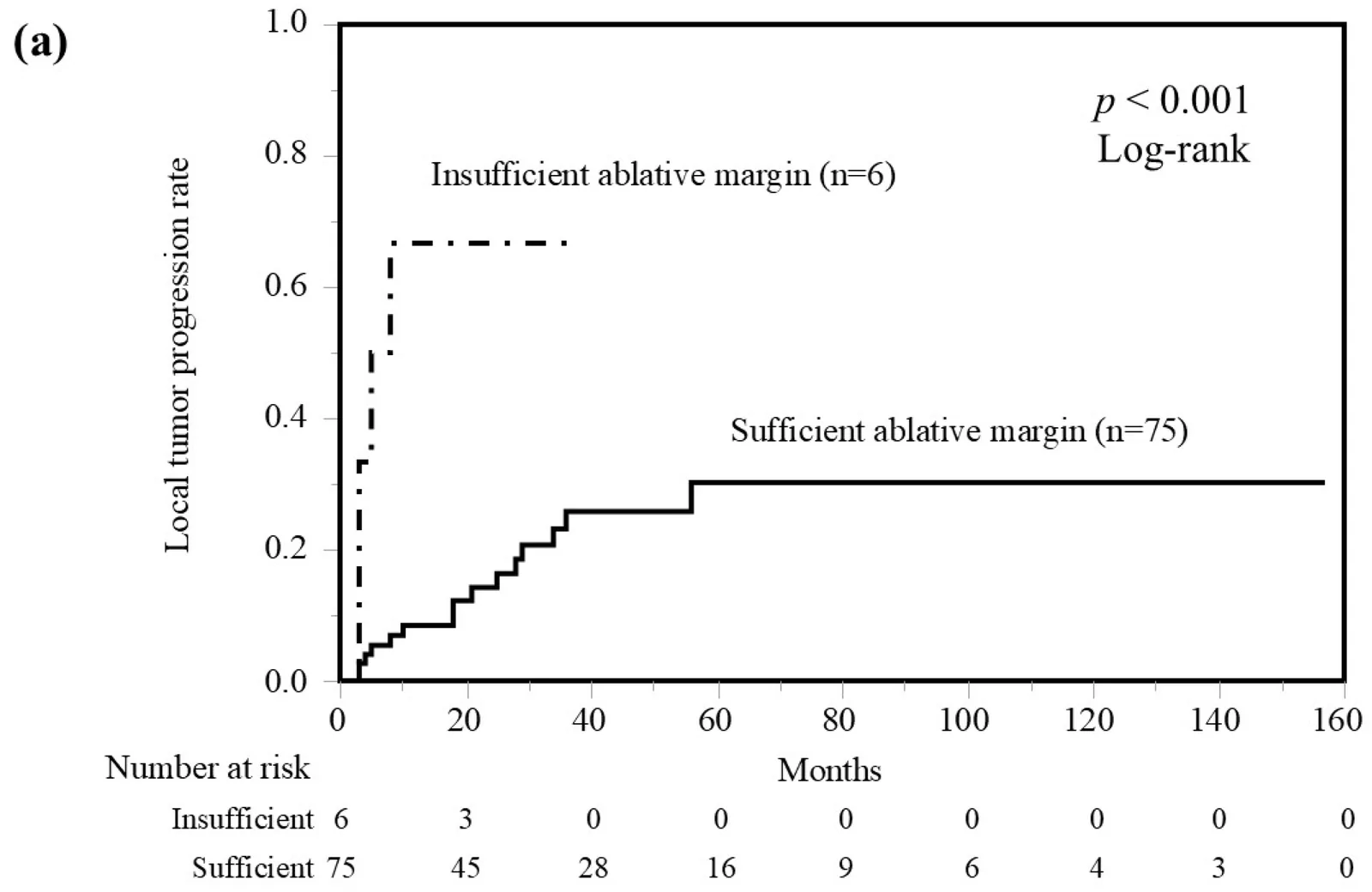

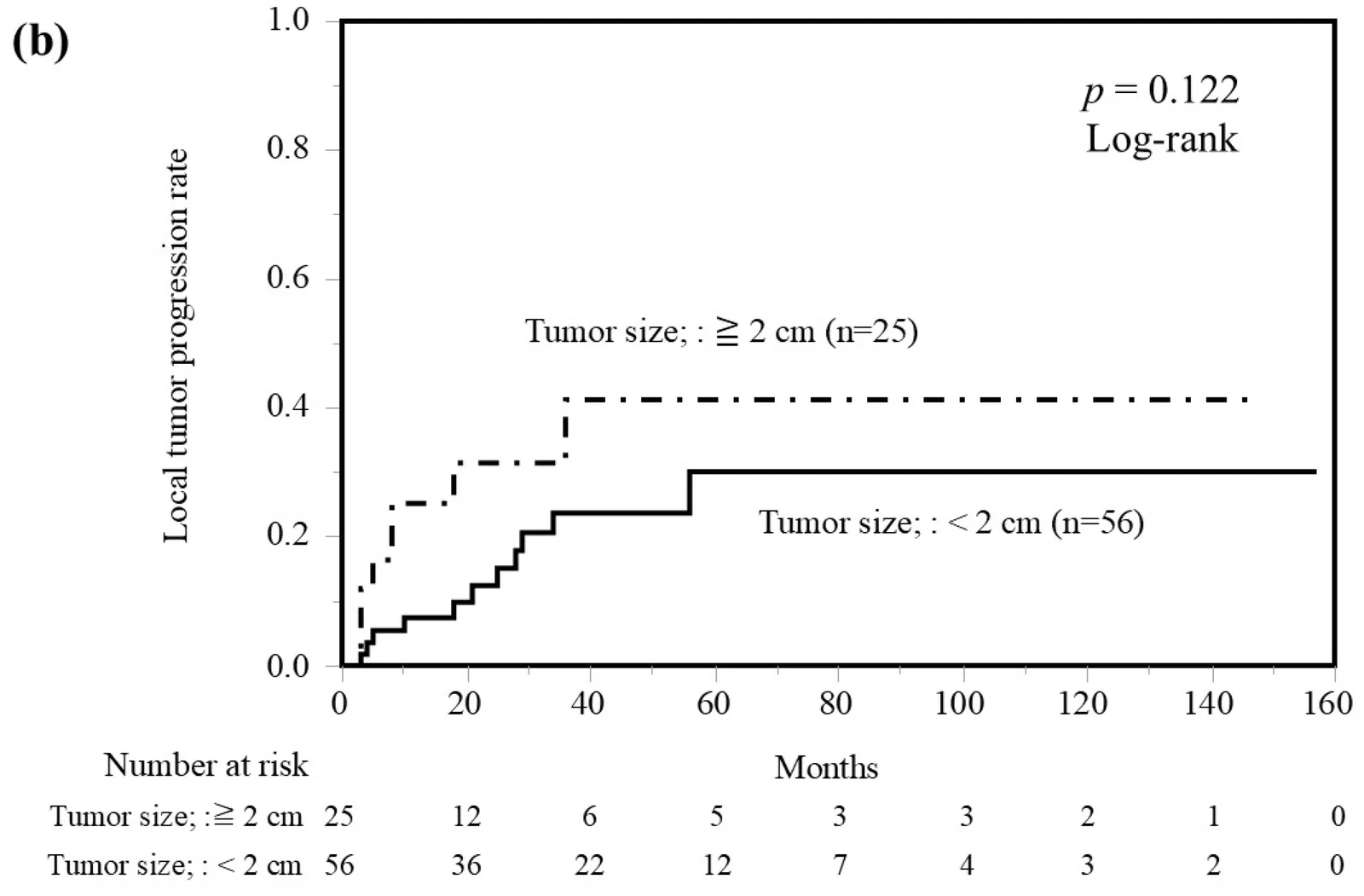

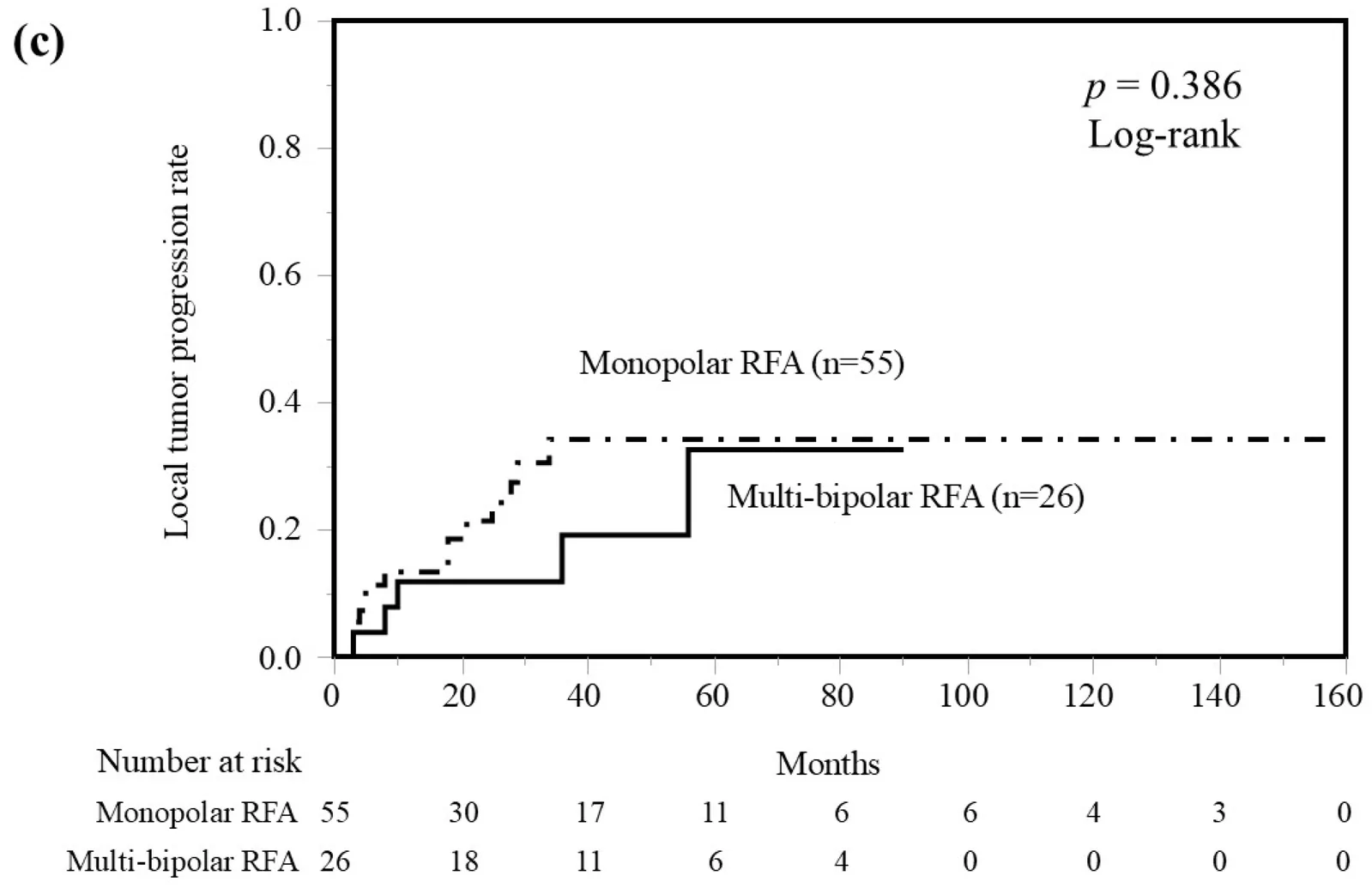

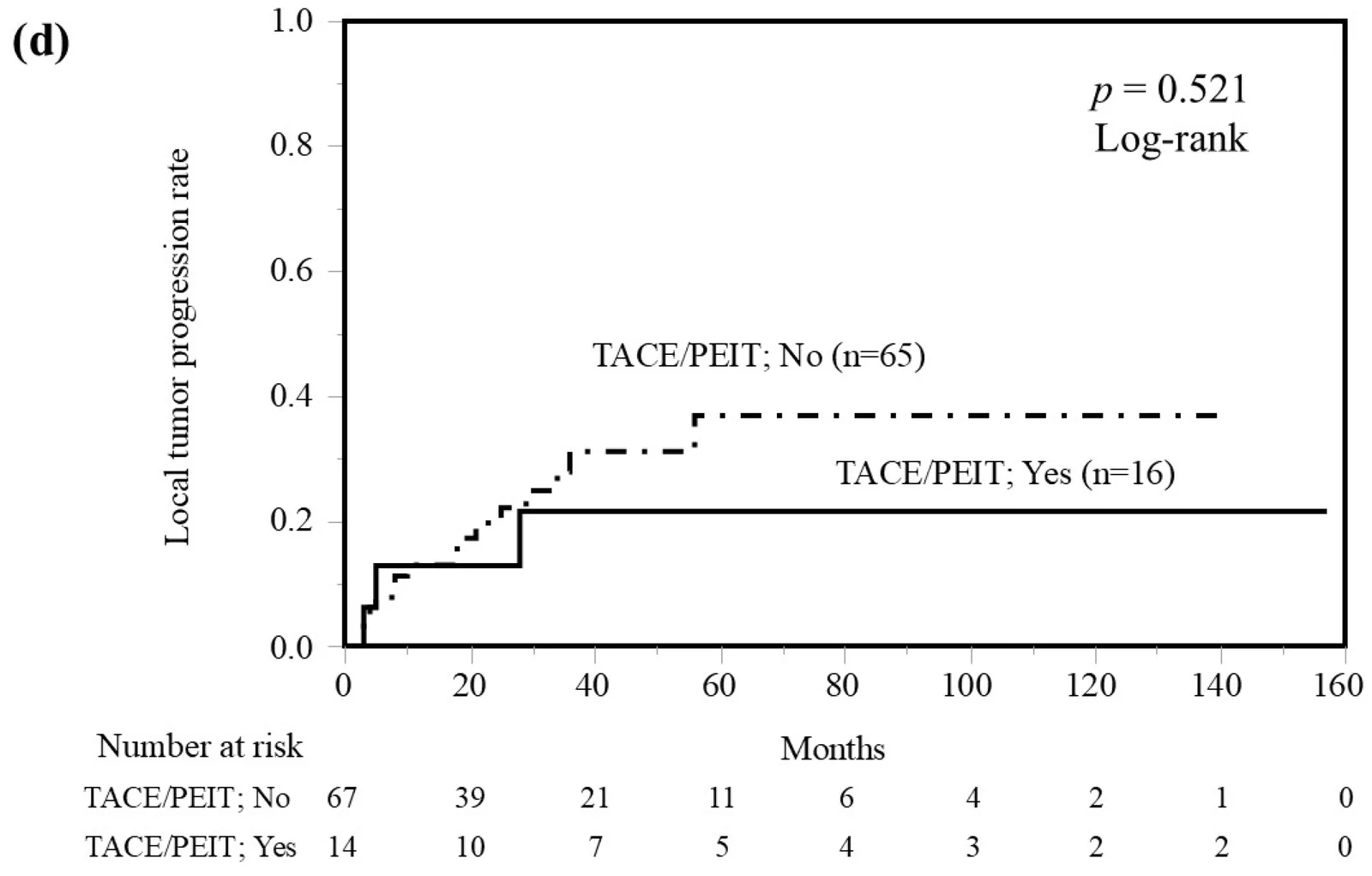
Kaplan–Meier curves for local tumor progression among several important factors. (**a**) ablative margin; sufficient (solid line) vs. insufficient (dashed line) (**b**) tumor size; <2 cm (solid line) vs. ≧2 cm (dashed line) (**c**) among different RFA devices; monopolar RFA (dashed line) vs. bipolar RFA (solid line) (**d**) additional treatment; TACE or PEIT; yes (solid line) vs. no (dashed line). Two patients had undergone both TACE and PEIT prior to RFA. Only “Sufficient ablative margin” showed a significant difference in LTP rate (*p* < 0.001, log-rank).

LTP was observed in 19 (23%) patients during the follow-up period. The cumulative LTP rates at 1, 3, 5, and 10 years were 13%, 29%, 33%, and 33%, respectively (Figure 3). The prognostic factors associated with LTP are shown in Figure 4. Among the examined variables, only an insufficient ablative margin was significantly associated with LTP, based on the log-rank test (*p* < 0.001). The factors contributing to local recurrence are listed in Table 3. In multivariate analysis, an insufficient ablative margin remained the only significant risk factor for LTP (hazard ratio [HR], 4.64; 95% confidence interval [CI], 1.14–14.9; *p* = 0.031).

### 3.4. Tumor Seeding and Adverse Events

**Figure 5:**
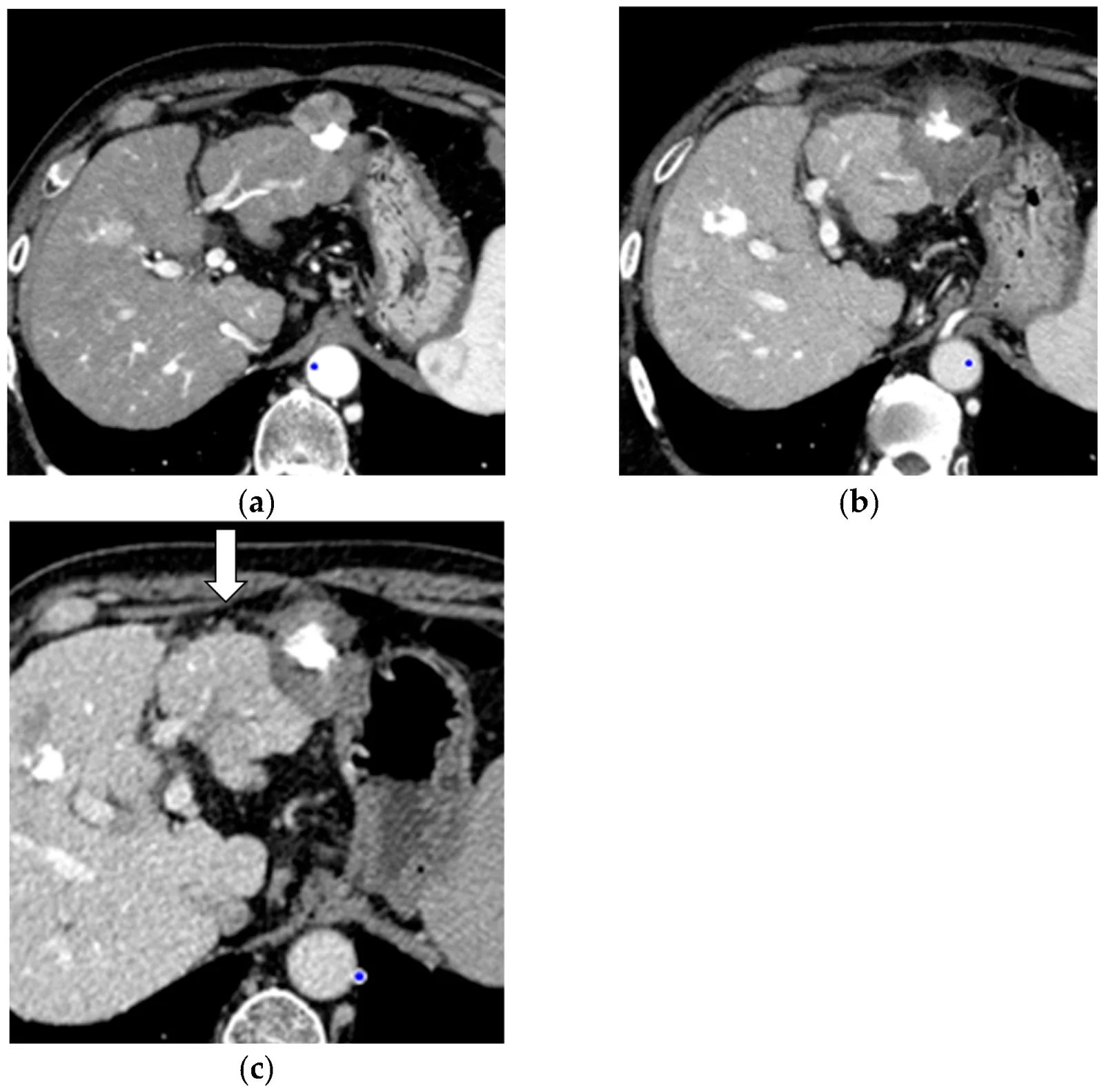
An 85-year-old male with HCC of 3.3 cm in diameter at the liver surface on segment 3 underwent RFA after TACE was performed. The RFA treatment was completed in a single session, during which the monopolar RFA electrode was inserted five times from different directions. (**a**) CT imaging before RFA. (**b**) CT imaging after RFA showed that a sufficient ablative area was acquired. (**c**) CT imaging after 3 months showed that seeding had appeared near the RFA scar (white arrow).

Tumor seeding was observed in only one case (1.2%). The case in which seeding occurred is shown in Figure 5. A 3.3 cm HCC located in the lateral lobe was treated with monopolar RFA. TACE was performed before RFA. As a result, RFA required one session with five needle insertions from multiple directions. Seeding was detected 3 months after the RFA treatment near the original lesion.

Adverse events are shown in Table 4. These included pleural effusion in 6 (7.4%), intraperitoneal bleeding in 5 (6.2%), abdominal pain in 3 (3.7%), portal vein thrombosis in 2 (2.5%), and sepsis in 1 patient (1.2%). No adverse events of Clavien–Dindo Grade III or higher were observed in our cohort. All adverse events were managed conservatively without the need for surgical or invasive intervention.

## 4. Discussion

The principal finding of this study was that an insufficient ablative margin was the only factor independently associated with LTP after RFA for subcapsular HCC. Although subcapsular location has traditionally been regarded as a technically challenging feature for percutaneous RFA, our findings suggest that achieving an adequate ablative margin may be more important for local tumor control than the subcapsular location itself.

Previous studies have identified subcapsular location as a factor associated with technical difficulty and treatment failure after percutaneous ablation. Consi et al. suggested the Ablation Difficulty Score (ADS), which was investigated according to clinical and radiological factors related to ablation failure, including size > 20 mm (*p* = 0.002), subcapsular location (*p* = 0.008), perivascular location (*p* = 0.024), isoechoic appearance (*p* = 0.008), non-cirrhotic liver (*p* = 0.009), and developed a scoring system related to LTP rates [12]. Han et al. reported that they compared the safety and efficacy of RFA for single HCCs ≤ 3 cm in subcapsular versus non-subcapsular locations using a propensity score matched analysis. Although the rate of technical effectiveness was the same (subcapsular vs. non-subcapsular; 98% vs. 99%, *p* = 0.315), rates of major complications and LTP differed significantly following first-line RFA treatment of single HCCs ≤ 3 cm in favor of the non-subcapsular locations [14]. These studies support the validity of the RFA difficulty score proposed by Consi et al. It is difficult to treat subcapsular HCC with RFA, both in terms of the technical procedure and poor treatment outcomes.

Generally, among the RFA treatments for early-stage HCC, several studies have shown that a margin of ablation ≥ 5 mm is important in reducing the local recurrence rate and improving local control. Nakashima et al. revealed that 59.1% of small HCCs ≤3.0 cm in diameter had micro-metastases within 5 mm of each micro-metastatic lesion and its primary HCC. Notably, among single nodular type HCC, micro-metastases were shown surrounding the tumor in 77.8% within 5 mm [15]. Okusaka et al. reported that microvascular invasion was observed in 17% of patients with tumors < 2 cm in diameter and 20% of patients with tumors 2 to 3 cm in diameter [16]. For these pathological reasons, ablation including a safety margin of the tumor at least more than 5 mm is required for RFA to achieve technical success [17,18,19,20]. Therefore, acquiring an adequate ablative margin (>5 mm) is critical for reducing the risk of LTP. However, this can be technically challenging for capsular lesions because of their proximity to vital structures. Although it is difficult to acquire an adequate ablative margin (>5 mm) for subcapsular HCC, various techniques have been used to improve treatment outcomes. Various reports have described the use of additional RFA techniques for the treatment of subcapsular HCC with RFA [9,21,22,23,24]. This study evaluated the use of artificial ascites, no-touch multi-bipolar RFA, TACE, and PEIT to obtain sufficient ablative margins in RFA for subcapsular HCC. Thus, laparoscopic RFA, MWA, or placement of sodium hyaluronate injection were not performed in this study and could not be evaluated.

Artificial ascites was used to physically separate the liver surface from the adjacent organs, thereby enhancing the safety margins. Toyoda et al. reported that it can be safely and effectively performed on subcapsular HCC by placing sodium hyaluronate on the liver surface, thereby preventing burn injuries to the abdominal wall or adjacent organs [21]. Kang et al. reported that long-term local tumor control and the risk of peritoneal seeding were comparable for RFA with or without artificial ascites when used as a first-line therapy for subcapsular HCC [9]. Moreover, emerging modalities such as the MWA system may offer complementary or alternative options for treating tumors in challenging locations. Zheng et al. reported that MWA resulted in better tumor control for subcapsular HCC within the Milan criteria. There was no difference in the incidence of major complications between the two groups [22]. However, RFA remains widely available and cost-effective, making it a valuable tool in interventional oncology armamentaria, particularly in resource-limited settings.

Morimoto et al. reported that multipolar laparoscopic RFA was effective in subcapsular HCC treatment by safely achieving a sufficient ablative margin [23]. All patients were alive with no LTP, and the 2-year local tumor progression-free survival rate was 100%. Laparoscopic treatment is considered effective; however, it requires general anesthesia and advanced surgical skills, making it less versatile than other treatment modalities.

Petit et al. reported that no-touch multi-bipolar RFA is a safe and effective treatment for subcapsular HCC not puncturable via the non-tumorous liver parenchyma [24]. A complete ablation was achieved in 57/58 patients (98.3%); the 1-, 2- and 3-year local tumor progression-free survival rates were 98%, 94% and 91%, respectively, and no patients had tumor seeding after no-touch multi-bipolar RFA treatment. Multi-bipolar RFA is considered effective; however, it is not suitable for treating all nodules. In regions where the liver parenchyma is thin, such as the lateral segment, puncturing may be challenging depending on the length of the electrode needle.

These findings have important implications for treatment selection. Although surgical resection may provide better local control in selected patients with small subcapsular HCC [25], many patients are unsuitable for surgery. In such cases, percutaneous RFA remains a viable minimally invasive option when safe tumor access and an adequate ablative margin can be achieved. Thus, subcapsular location alone should not preclude RFA. Careful treatment planning, additional ablation when necessary, and early post-ablation imaging when a margin of <5 mm is suspected may help reduce LTP.

Notably, the risk of peritoneal seeding, although rare, underscores the importance of avoiding tumor rupture during electrode insertion and ablation for subcapsular HCC. In the current study, a case of HCC protruding 3 cm into the lateral region that experienced dissemination after TACE treatment was deemed insufficient and RFA treatment was added. Owing to the large tumor diameter, five treatment sessions were required from multiple directions, and as a result, dissemination occurred three months later. There have been various reports on tumor seeding associated with RFA treatment. Seeding is a potential adverse event, although it should be avoided in RFA therapy, and the incidence of seeding is generally reported to range from 0.14% to 5.1% [26,27,28,29,30,31,32]. Jaskolka et al. reported that needle tract seeding occurred in eight of 200 patients (4.0%), representing 2.7% per treatment session and 2.7% per lesion. Seeding was more frequent in subcapsular tumors, patients who underwent multiple RFA sessions, and those who underwent multiple punctures [26]. Llovet et al. reported four of 32 cases of seeding in their early report of RFA treatment for subcapsular HCC [27]. In a meta-analysis, Kim et al. reported 7.9% to 36% seeding of subcapsular HCC without tract ablation, and 0% with careful tract ablation [10]. In the current study, tract ablation was performed in all cases, and seeding occurred in only one case (1.2%), suggesting that seeding was not a high incidence for the treatment of subcapsular HCC in patients who underwent RFA with sufficient tract ablation.

This study has several strengths. It specifically evaluated percutaneous RFA for subcapsular HCC, including LTP, complications, and tumor seeding, over a 13-year period. The use of contemporary techniques, including artificial ascites and no-touch multi-bipolar RFA, allowed treatment to be tailored to tumor characteristics. Importantly, the study identified the ablative margin as a potentially modifiable factor associated with LTP, providing a practical target for improving local tumor control.

This study has several limitations, mostly related to its retrospective and non-comparative design. Furthermore, the number of cases was smaller than that reported in previous studies focusing on the same topic. Moreover, we cannot directly compare the technical difficulty or clinical outcomes of RFA between subcapsular and non-subcapsular HCC based on our own data. Second, MWA was not performed in this study. Recently, several articles reported that MWA was effective for HCC treatment because MWA promised to acquire an oval ablation area and did not influence the cooling effect from the vessels [6,33,34]. Moreover, MWA is useful for subcapsular HCC [23]. Third, the long follow-up period makes it difficult to attribute outcomes to the procedure itself versus technological improvements. Additionally, advanced techniques, such as monopolar RFA or the no-touch multi-bipolar RFA procedure, were not introduced simultaneously during the study period and may have influenced the outcome of this study. Fourth, the decision to use adjunctive techniques such as pre-RFA TACE for tumors > 2 cm, PEIT for protruding tumors, and artificial ascites was not protocolized but left to the operator’s discretion, potentially introducing confounding bias. Finally, in the multivariate Cox regression analysis, only six cases showed “insufficient ablative margin” resulting in a wide confidence interval range (1.14–14.9) and raising the statistical risk of overfitting. Therefore, further large-scale, multicenter prospective studies are necessary.

## 5. Conclusions

Achieving an adequate ablative margin of at least 5 mm is crucial, regardless of tumor location, the number of treatment sessions, or the type of RFA device used. To minimize the risk of LTP after RFA for subcapsular HCC, additional ablation should be performed when the ablation zone does not completely encompass the tumor after a single session. Furthermore, if post procedural CT demonstrates an insufficient ablative margin, additional RFA should be performed promptly.

Tumor seeding was observed in only one case, which may reflect the routine use of tract ablation during electrode withdrawal in all procedures. Further prospective multicenter studies are warranted to validate these findings.

## Figures and Tables

**Table 1:** The baseline characteristics of the patients.

Factors	Values
Age (years) #	74 (46, 93)
Gender $	
Male	59 (73)
Female	22 (27)
Child Pugh classification $	
A	68 (84)
B	13 (16)
Naïve HCC $	
Yes	21 (26)
No	60 (74)
Tumor diameter (cm) #	1.5 (0.5, 3.7)
Tumor location (hepatic segment) $	
1	0 (0)
2	3 (4)
3	16 (20)
4	9 (11)
5	27 (33)
6	9 (11)
7	6 (7)
8	11 (14)
AFP (ng/mL) #	6.9 (1.1, 280)
DCP (mAU/mL) #	27 (10, 3755)

$; Number of the Patients (%). #; Median (minimum, Maximum).

**Table 2:** The results associated with RFA treatment in the present study.

Factors	Values *n* (%)
RFA devices	
Monopolar RFA	55 (68)
Multipolar RFA	26 (32)
Performed TACE before RFA therapy	
Yes	10 (12)
No	71 (88)
Performed PEIT before RFA therapy	
Yes	7 (9)
No	74 (91)
Artificial ascites	
Yes	26 (32)
No	55 (68)
Insertion sessions	
One	34 (42)
Two	33 (41)
Three	12 (15)
Four	0 (0)
Five	2 (2)
Sufficient ablative margin (>5 mm)	
Yes	75 (93)
No	6 (7)
Local tumor progression	
Yes	19 (23)
No	62 (77)

RFA; Radiofrequency ablation, TACE; Transcatheter arterial chemoembolization, PEIT; Percutaneous ethanol injection therapy.

**Table 3:** The factors associated with local tumor progression after radiofrequency ablation for patients with HCC at the hepatic capsule.

	Univariate Analysis		Multivariate Analysis	
Factors	HR (95% CI) &	*p* Value $	HR (95% CI) &	*p* Value $
Age (years) ≥ 75	1.98 (0.77–5.04)	0.146	1.65 (0.59–4.61)	0.340
Recurrence HCC	1.36 (0.45–4.12)	0.580		
Child-Pugh grade B	1.94 (0.64–5.92)	0.243	1.99 (0.57–6.89)	0.277
Tumor size (cm) ≥ 2.0	2.05 (0.82–5.14)	0.122	1.54 (0.59–4.03)	0.371
Localization: Right lobe	1.40 (0.55–3.57)	0.479		
AFP (ng/mL) ≥ 10	0.71 (0.27–1.88)	0.495		
DCP (mAU/mL) ≥ 40	1.03 (0.40–2.65)	0.959		
Monopolar RFA	1.56 (0.56–4.35)	0.393	1.42 (0.49–4.14)	0.522
TACE: not performed	1.03 (0.24–4.50)	0.959		
PEIT: not performed	1.09 (0.25–4.75)	0.909		
Artificial ascites; Yes	1.79 (0.72–4.47)	0.210	1.93 (0.73–5.12)	0.185
Insertion session: One	0.81 (0.32–2.07)	0.666		
Insufficient ablative margin	5.96 (1.93–18.4)	0.002	4.64 (1.14–14.9)	0.031

&: Relative risks were calculated by comparing classes with Cox regression analysis. $: *p*-values were obtained by using Cox regression analysis. HR: Hazard ratio, CI: confidence interval.

**Table 4:** Adverse events associated with RFA treatment in the present study.

Adverse Events	Value (*n* = 81) *n* (%)
Pleural effusion	6 (7.4)
Intraperitoneal Bleeding	5 (6.2)
Abdominal pain	3 (3.7)
Portal vein thrombosis	2 (2.5)
Sepsis	1 (1.2)

## Data Availability

Data from this study may be provided by the corresponding author upon reasonable request.

## Abbreviations

The following abbreviations are used in this manuscript:

HCC	Hepatocellular carcinoma
RFA	Radiofrequency ablation
MWA	Microwave ablation
HR	Hazard ratio
TACE	Transcatheter arterial chemoembolization
PEIT	Percutaneous ethanol injection therapy
AFP	Alpha-fetoprotein
DCP	Des-gamma-carboxy prothrombin
CT	Computed tomography
MRI	Magnetic resonance imaging
